# First-principles calculations of the epsilon phase of solid oxygen

**DOI:** 10.1038/s41598-019-45314-9

**Published:** 2019-06-19

**Authors:** Le The Anh, Masahiro Wada, Hiroshi Fukui, Tsutomu Kawatsu, Toshiaki Iitaka

**Affiliations:** 1 Computational Astrophysics Laboratory, RIKEN, 2-1 Hirosawa, Wako, Saitama 351-0198 Japan; 2 Graduate school of Material Science, University of Hyogo, 3-2-1 Kouto, Kamigori, Hyogo 678-1297 Japan; 3 Centre for Computational Physics, Institute of Physics, Vietnam Academy of Science and Technology, 10 Dao Tan, Ba Dinh, Hanoi Vietnam

**Keywords:** Electronic properties and materials, Magnetic properties and materials

## Abstract

The crystal, electronic and magnetic structures of solid oxygen in the epsilon phase have been investigated using the strongly constrained appropriately normed (SCAN) + rVV10 method and the generalized gradient approximation (GGA) + vdW-D + U method. The spin-polarized SCAN + rVV10 method with an 8-atom primitive unit cell provides lattice parameters consistent with the experimental results over the entire pressure range, including the epsilon-zeta structural phase transition at high pressure, but does not provide accurate values of the intermolecular distances *d*_1_ and *d*_2_ at low pressure. The agreement between the intermolecular distances and the experimental values is greatly improved when a 16-atom conventional unit cell is used. Therefore, the SCAN + rVV10 method with a 16-atom unit cell can be considered the most suitable model for the epsilon phase of solid oxygen. The spin-polarized SCAN + rVV10 model predicts a magnetic phase at low pressure. Since the lattice parameters of the predicted magnetic structure are consistent with the experimental lattice parameters measured at room temperature, our results may suggest that the epsilon phase is magnetic even at room temperature. The GGA + vdW-D + U (with an ad hoc value of *U*_*eff*_ = 2 eV at low pressure instead of the first-principles value of *U*^*lr*^_*eff*_ ~ 9 eV) and hybrid functional methods provide similar results to the SCAN + rVV10 method; however, they do not provide reasonable values for the intermolecular distances.

## Introduction

Oxygen, the third most abundant element in the universe^1^, makes up approximately 46% of the components of the Earth’s crust and 21% of the atmosphere. Oxygen can exist not only on the Earth’s surface but also deep inside the mantle layers. According to molecular theory, an oxygen molecule O_2_ has two unpaired electrons that make O_2_ an active substance. Oxygen can therefore easily form compounds with many elements in the Earth’s mantle. The depth of the Earth’s mantle ranges from approximately 100 km to 3000 km, and the corresponding pressure ranges from approximately a few GPa up to 150 GPa. Under such high pressure, O_2_ gas must be solidified. To understand O_2_ and its compounds in the mantle, the structural evolution, electronic and magnetic properties of molecular oxygen under high pressure are investigated in this article.

The O_2_ molecule under high pressure has been widely studied over many decades^2–11^. It was shown in a neutron diffraction experiment^12^ that solid oxygen transforms from the anti-ferromagnetic delta phase to the non-magnetic epsilon phase at 7.6~8 GPa, while recent generalized gradient approximation (GGA) + U calculations also suggested a low-pressure anti-ferromagnetic epsilon phase of oxygen (from ~10 GPa up to 20 GPa) before it completely transforms into the non-magnetic epsilon phase^13,14^. To examine the ability of state-of-the-art density functional theory (DFT) methods to predict the crystal and electronic structures of solid oxygen, the results of two quasi-local density functionals combined with van der Waals (and Hubbard U) corrections, i.e., the GGA + vdW-D + U method and strongly constrained appropriately normed (SCAN) + rVV10 method^15^, are compared with experimental results^9,16^. The GGA + vdW-D + U method uses a GGA (PBE^17^) functional combined with a semi-empirical vdW interaction^18^ and a Hubbard U correction for the on-site Coulomb interaction between the *p* orbitals of the oxygen atom. The SCAN + rVV10 method^15^ uses a meta-GGA SCAN functional, which is accurate for short and intermediate ranges, combined with a first-principles long-range van der Waals interaction (rVV10). Thus, this approach is expected to accurately reproduce the structures and electronic properties of molecular crystalline solid oxygen. The SCAN + rVV10 method does not require a Hubbard U correction since it describes the on-site Coulomb interaction with sufficient accuracy.

## Computational Methods

DFT calculations were performed using the Quantum ESPRESSO package^19^ with norm-conserving pseudopotentials^20^ and the VASP package^21^ with a projector augmented wave (PAW) pseudopotential. The number of k points in the irreducible Brillouin zone was equal to 88 (5 × 5 × 7 Monkhost-Pack sampling). The kinetic energy cut-off for the wavefunctions was set at 150 Ry with a 10^−8^ Ry total energy convergence for one self-consistent field (SCF) cycle. Variable-cell optimization was carried out to optimize both the lattice parameters and atomic coordinates. A primitive cell consisting of 8 oxygen atoms was used unless otherwise noted. The Broyden–Fletcher–Goldfarb–Shanno (BFGS) algorithm^22^ was used for optimization of both the ion positions and unit cell vectors in compression. The pressure was increased by 10 GPa from 10 GPa to 140 GPa. The convergence threshold of the forces for ionic minimization was set at 5 × 10^−4^ a.u.

To investigate the effects of various conventional functionals, we carry out our calculations for the local density approximation (LDA), Becke-Lee-Yang-Parr (BLYP), Perdew-Burke-Ernzerhof (PBE) and meta-GGA (M06L) functionals. The meta-GGA (M06L) functional predicts the epsilon-zeta transitional pressure (P_T_) to be 30 GPa with inaccurate lattice parameters compared to the experimental parameters. The BLYP and PBE functionals are the best among these functionals, showing the transition at 40 GPa with better lattice parameters. Moreover, we consider both semi-empirical^18^ and non-local van der Waals functionals^23,24^. We find that the van der Waals functionals give similar results to the GGA (PBE) without the van der Waals prediction (transitional pressure of P_T_ = 40 GPa), while the semi-empirical GGA + vdW-D method^18^ (Grimme potential) results in a small improvement (P_T_ = 50 GPa) with lattice parameters very close to those of the conventional GGA (PBE). The combination of the van der Waals functionals and the Hubbard U correction shows no improvement over the GGA + U method only (P_T_ = 70 GPa). While the combination of semi-empirical vdW-D^18^ and Hubbard U shows a small improvement with P_T_ = 80 GPa, the lattice parameters calculated with vdW-D + U and those with Hubbard U are very similar^25^. The effect of the van der Waals interaction, therefore, is small compared to the effect of the Hubbard U correction. The details of the comparison between different functionals are mentioned in the Supplementary Materials and reference^25^. In this study, we use the semi-empirical vdW-D^18^ (Grimme potential) functional only and vary the value of the constant *U*_*eff*_ to investigate the effect of the Hubbard correction. For results with various conventional functionals, please refer to the Supplementary Materials. For the Hubbard term, we use two methods: gradually varying the values of *U* and the first-principles rotationally invariant scheme of Cococcioni^26^, which can uniquely determine the value of *U*.

### Structural models

**Figure 1:**
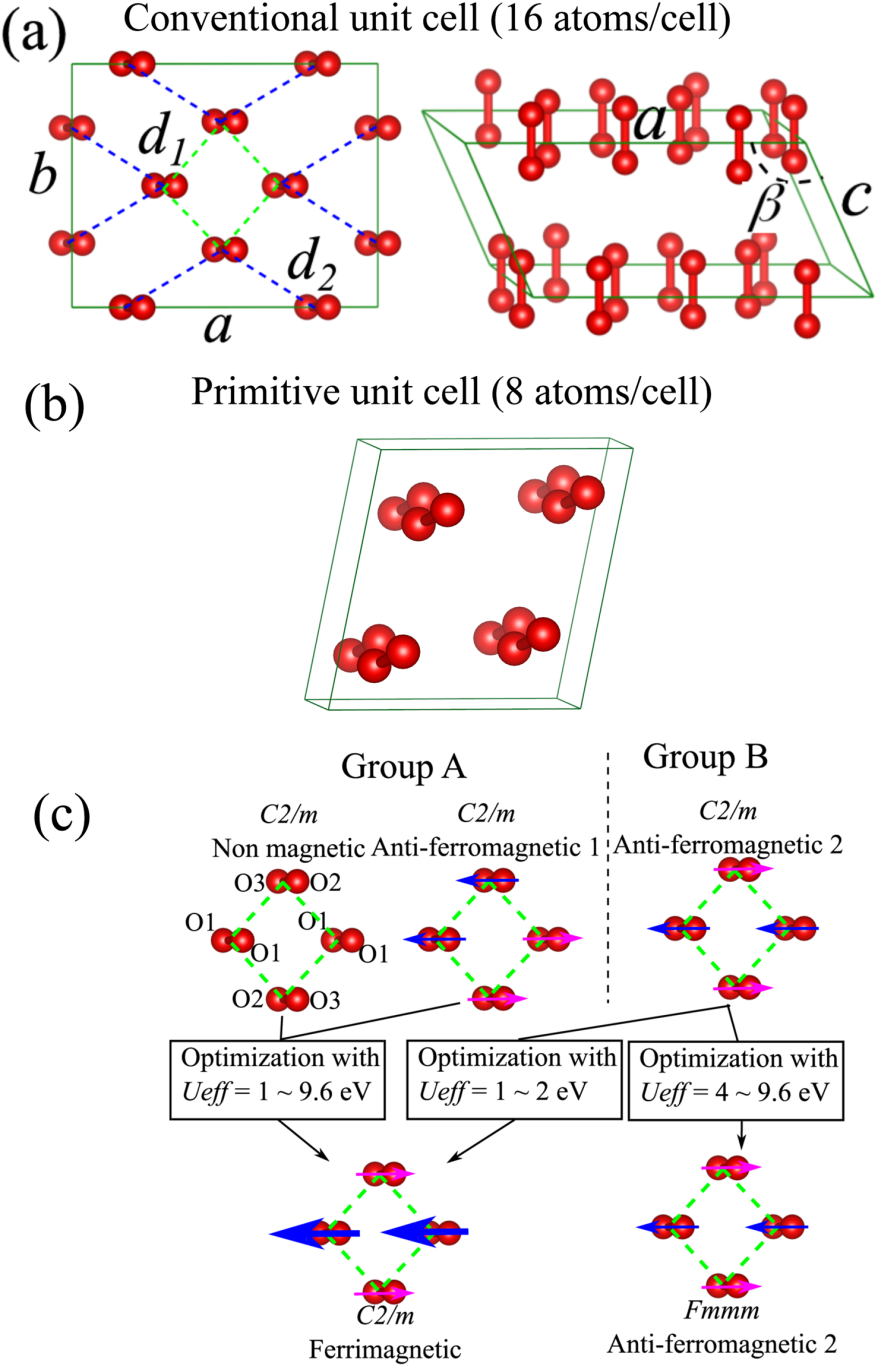
(**a**) Top and birds-eye views of the conventional unit cell (16 atoms/cell) of the epsilon phase with *C2/m* symmetry. The intermolecular distances *d*_1_ and *d*_2_ are also defined. (**b**) The primitive unit cell (8 atoms/cell). (**c**) The optimized magnetic structures are either *C2/m* ferrimagnetic or *Fmmm* anti-ferromagnetic depending on the value of *U*_*eff*_.

In Fig. 1(a), we show top and birds-eye views of the conventional unit cell of the epsilon phase and its lattice parameters, where the angle *β* between the *a* and *c* axes is larger than 90 degrees and the other angles equal 90 degrees. The intermolecular distances *d*_1_ and *d*_2_ are also defined as the distances between the centres of the O_2_ molecule in an (O_2_)_4_ cluster and between (O_2_)_4_ clusters as indicated in Fig. 1(a). The crystal structure has *C2/m* symmetry in which four O_2_ molecules gather to form an (O_2_)_4_ cluster^8^. The primitive cell which consists of 8 atoms is shown in Fig. 1(b). There are three symmetrically inequivalent atoms, O1, O2, and O3, in the *C2/m* structure, as shown in Fig. 1(c), where the initial spin configurations for the spin-polarized calculations are labelled *Non-magnetic*, *Anti-ferromagnetic* 1 and *Anti-ferromagnetic* 2. We classify the initial configurations into group A (*Non-magnetic* and *Anti-ferromagnetic 1*) and group B (*Anti-ferromagnetic* 2). The final structures after structural optimization in the spin-polarized calculations are either ferrimagnetic or anti-ferromagnetic depending on the initial spin configurations and the value of the Hubbard energy as indicated in Fig. 1(c). Since the total magnetization becomes zero for both the anti-ferromagnetic and non-magnetic configurations, the absolute magnetization M is defined to estimate the degree of spin polarization in the ferrimagnetic and anti-ferromagnetic phases by:

1 $${\boldsymbol{M}}={\int }^{}|{{\boldsymbol{n}}}_{{\boldsymbol{up}}}(\overrightarrow{{\boldsymbol{r}}})-{{\boldsymbol{n}}}_{{\boldsymbol{down}}}(\overrightarrow{{\boldsymbol{r}}})|{{\boldsymbol{d}}}^{3}\overrightarrow{{\boldsymbol{r}}}$$

where ${{\boldsymbol{n}}}_{{\boldsymbol{up}}}(\overrightarrow{{\boldsymbol{r}}})\,\,$and ${{\boldsymbol{n}}}_{{\boldsymbol{down}}}(\overrightarrow{{\boldsymbol{r}}})\,\,$are spin-up and spin-down densities, respectively.

## Results and Discussion

### Non-spin-polarized calculations (with constant *U*_*eff*_)

**Figure 2:**
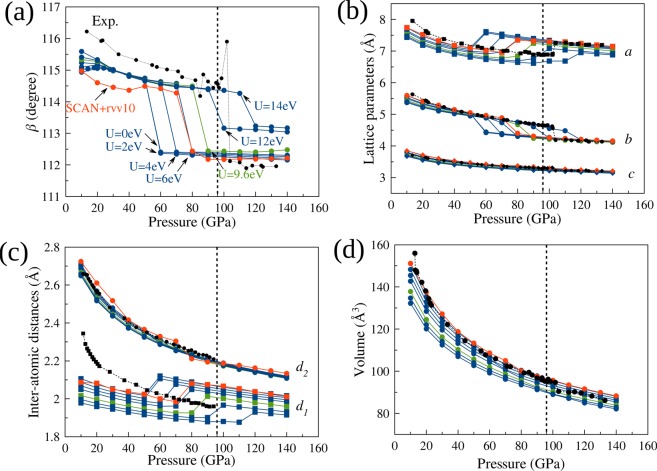
(**a**–**c**) Optimized *β* angle, *a, b, and c* lattice parameters, and the intermolecular distances *d*_1_ and *d*_2_ calculated using GGA + vdW-D + U with *U*_*eff*_ = 0, 2, 4, 6, 9.6, 12, and 14 eV in comparison with those for SCAN + rVV10 and experimental data adopted from Weck *et al*.^9^ and Fujihisha *et al*.^16^. The value *U*^*lr*^_*eff*_ = 9.6 eV was calculated using the first-principles linear response method^26^. (**d**) The volume of the unit cell in comparison with experiments^9^ (black points). The vertical dashed lines present the epsilon-zeta transition at 96 GPa in the experiment^9^.

Figure 2(a–c) shows the calculated lattice parameters *β, a, b*, and *c*, and the intermolecular distances *d*_*1*_ and *d*_2_ calculated using the GGA + vdW-D + U method with *U*_*eff*_ = 0, 2, 4, 6, 9.6, 12, and 14 eV in comparison with those for the SCAN + rVV10 method and the experimental data adopted from the X-ray diffraction measurement by Weck *et al*.^9^ (a, b) and Fujihisha *et al*.^16^ (c). In our calculations, we manually increase the value of *U*_*eff*_ from 0 eV to 14 eV. We call this the constant *U*_*eff*_ method. In addition, we calculate *U*^*lr*^_*eff*_ from the first-principles linear response method for each inequivalent atom at 10 GPa. The values of *U*^*lr*^_*eff*_ for each inequivalent atom are very similar, and the average value of *U*^*lr*^_*eff*_ is approximately 9.6 eV. We consider this value as the result of the first-principles linear response method and apply this value at various pressures in increments of 10 GPa in order to compare the results with the constant *U*_*eff*_ method with *U*_*eff*_ = 0, 2, 4, 6, 12, and 14 eV. As *U*_*eff*_ increases from 0 eV to 9.6 eV and 14 eV, the epsilon-zeta transition pressure increases from 50 GPa to 90 GPa and 110 GPa. Therefore, *U*_*eff*_ should be between 9.6 eV and 14 eV to be consistent with the experimental transition pressure of 96 GPa. This fact demonstrates that the linear response method, which gives *U*^*lr*^_*eff*_ ~ 9.6 eV, works well in the high-pressure regime.

In general, both the GGA + vdW-D (*U*_*eff*_ = 0 eV) and GGA + vdW-D + U calculations underestimate the lattice parameters of the epsilon phase at low pressure. As a result, the calculated volume of the unit cell for the epsilon phase is underestimated at low pressure, as indicated in Fig. 2(d). The results from the SCAN + rVV10 calculation are closer to the experimental data than those from the GGA + vdW-D and GGA + vdW-D + U calculations. In particular, the SCAN + rVV10 and GGA + vdW-D + U calculations with *U*_*eff*_* ≤ *2 eV give the most consistent volumes at low pressure. This suggests that *U*_*eff*_ should be smaller than 2 eV at low pressure (≤20 GPa) and approximately 9.6 eV at higher pressure.

### Non-spin-polarized calculations (with updated first-principles *U*^*lr*^_*eff*_)

**Figure 3:**
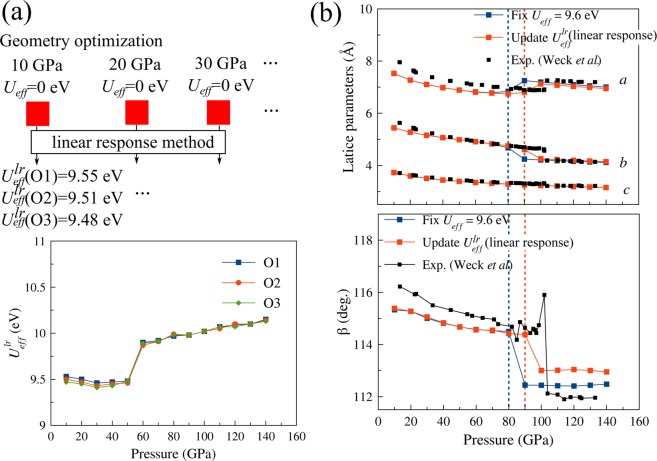
(**a**) Upper: flow chart to calculate *U*^*lr*^_*eff*_ at pressures in increments of 10 GPa. Lower: *U*^*lr*^_*eff*_ at different pressures and different oxygen atom sites. (**b**) Upper: lattice parameters *a, b, c* and the angle *β* calculated with fixed (constant) *U*_*eff*_ = 9.6 eV, with the first-principles value of *U*^*lr*^_*eff*_ updated from the linear response method^26^ in comparison with the experimental data^9^.

To determine the correct values of the Hubbard *U* parameter at each pressure, we performed a first-principles linear response calculation of the Hubbard U parameter for the structure optimized at each pressure with the GGA + vdW-D method. In Fig. 3(a), the first-principles *U*^*lr*^_*eff*_ values for the inequivalent atoms are plotted as a function of pressure. The values exhibit a very small variation with respect to the inequivalent atoms and exhibit a small pressure dependence ranging from 9.5 eV (P ≤ 50 GPa) to 10.2 eV (P > 50 GPa) with a jump due to the epsilon-zeta structural transition at 50 GPa calculated with the GGA + vdW-D method. Since this jump is still much smaller than the absolute value of *U*, the pressure-dependent first-principles *U* may be approximated by the constant *U*^*lr*^_*eff*_ = 9.6 eV for all inequivalent atoms at all pressures. The convergence with the cell size was confirmed by a calculation with a larger cell. Interestingly, this first-principles result turns out to be inconsistent with the empirical estimation in the previous paragraph where *U*_*eff*_ should be smaller than 2 eV at low pressure (≤20 GPa) and approximately 9.6 eV at higher pressure. This problem will be further discussed in the paragraphs related to the spin-polarized calculation.

As shown in Fig. 3(b), the lattice parameters calculated with the empirical constant *U*_*eff*_ = 9.6 eV and those calculated with the first-principles *U*^*lr*^_*eff*_ values are very similar. The first-principles *U*^*lr*^_*eff*_ predicts a transition pressure of 90 GPa, which is closer to the experimental pressure than the transition pressure of 80 GPa predicted with a constant *U*_*eff*_ = 9.6 eV. In the following, the results with the first-principles *U*^*lr*^_*eff*_ are used for the GGA + vdW-D + U calculation unless otherwise stated.

**Figure 4:**
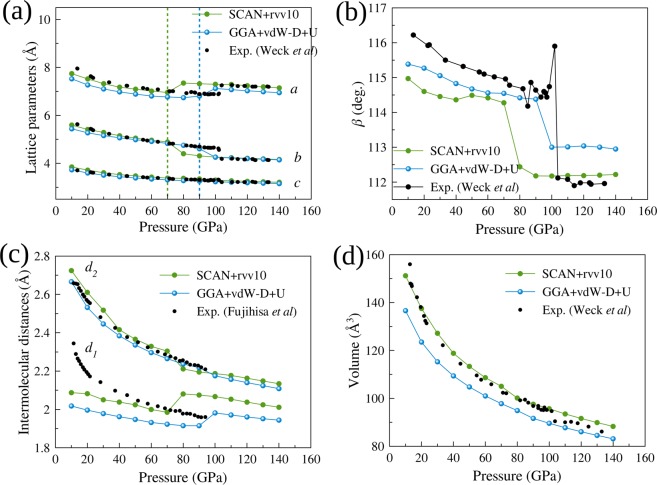
(**a**–**d**) Optimized *a, b*, and *c* lattice parameters, *β* angle, intermolecular distances *d*_1_ and *d*_2_ and the unit cell volume calculated with the non-spin-polarized SCAN + rVV10 and GGA + vdW-D + U methods in comparison with experimental data^9,16^.

In Fig. 4(a–d), we show the lattice parameters *a, b*, and *c*, the angle *β*, the intermolecular distances *d*_1_ and *d*_2_, and the unit cell volume calculated using the SCAN + rVV10 and GGA + vdW-D + U methods in comparison with the experimental measurements^9,16^. The parameters from the GGA + vdw-D + U method are close to those from the SCAN + rVV10 method, although the SCAN + rVV10 method gives parameters closer to the experimental values overall. In general, the non-spin-polarized GGA + vdW-D + U (*U*^*lr*^_*eff*_ ~ 9.6 eV) calculation can predict reasonable lattice parameters of the epsilon phase of solid oxygen at pressures above 20 GPa and can even predict the epsilon-zeta transition pressure at 90 GPa, which is very close to the experimental value of 96 GPa. Interestingly, below 20 GPa, both the GGA + vdW-D + U and SCAN + rVV10 calculations significantly underestimate *d*_1_. This problem will be discussed in a later part of the paper.

**Figure 5:**
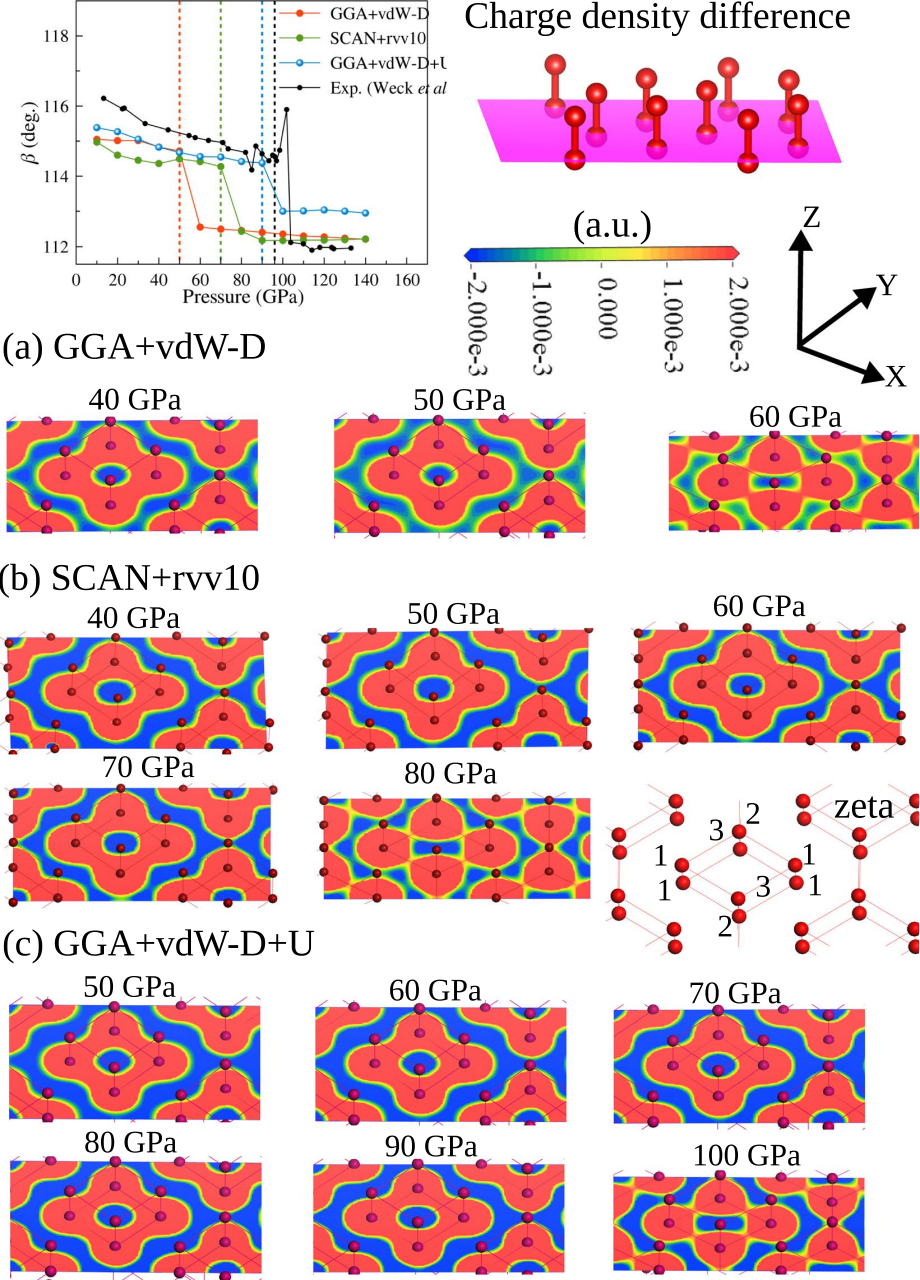
The evolution of cross-sections of the charge density difference in the *ab* plane: (**a**) GGA + vdW-D, (**b**) SCAN + rVV10, and (**c**) GGA + vdW-D + U. The position of the cross-section is illustrated by the purple plane. The transition from the epsilon phase to the zeta phase can be clearly observed.

To investigate the evolution of the chemical bonds inside the (O_2_)_4_ structure and between (O_2_)_4_ structures in compression, we consider the charge density difference (CDD) as the charge density minus the superposition of atomic densities. A positive CDD indicates that the electron density increases, and a negative CDD indicates that the electron density decreases with respect to the charge density of isolated individual atoms. Therefore, the CDD can indicate how the chemical bonds form and evolve. Figure 5 shows a cross-section of the CDD in the *ab* plane (the purple plane) in three cases: (a) GGA + vdW-D (*U*_*eff*_ = 0 eV), (b) SCAN + rVV10, and (c) GGA + vdW-D + U (with first-principles *U*^*lr*^_*eff*_). Compared to the GGA + vdW-D calculation, both the SCAN + rVV10 and GGA + vdW-D + U calculations result in an increase in the electron density inside the (O_2_)_4_ cluster and a decrease in the electron density between the (O_2_)_4_ clusters, which means that the electron is more localized within the (O_2_)_4_ region. This means that the enhancement of the localization of *p* orbitals using the Hubbard *U* correction is necessary for describing the electronic structure of solid oxygen. Interestingly, in the zeta phase, the electron charge around O2 and O3 connects to those of the neighbouring (O_2_)_4,_ while O1 is still isolated from the neighbouring (O_2_)_4_. These results suggest that the metallization of epsilon phase may start by the connection of electron charge density between either O2 or O3 of an (O_2_)_4_ cluster to O2 or O3 of neighboring cluster and completely finish when the electron charge density spreads to all atoms O1, O2, and O3. That indicates the metallization should occur gradually. Our results also confirm the experimental results^8,16^ that epsilon phase has (O_2_)_4_ structure and suggest the *C2/m* zeta phase^11^.

In summary, the non-spin-polarized GGA + vdW-D + U method can predict the direct transition from the epsilon phase to the zeta phase for the first time due to the enhancement of the electron density localization at 90 GPa with the values of *U*^*lr*^_*eff*_ (~ 9.6 eV) obtained from the first-principles linear response method^26^. The result from the first-principles SCAN + rVV10 functional is 70 GPa. The GW calculations^27,28^ predicted an insulator-metal transition at ~100 GPa (but a structural transition at 50 GPa). This value of *U*^*lr*^_*eff*_ (~9.6 eV) is not too high for the GGA + vdW-D + U method if we compare other studies in which DFT + U in combination with van der Waals density functionals reported that *U*_*eff*_ = 5 eV and *U*_*eff*_ = 12 eV are needed for the prediction of the lattice parameters of the alpha phase of solid oxygen using the revPBE and optB86b exchanges, respectively^29^. At low pressure (below 20 GPa), all the DFT calculations underestimate all of the lattice parameters, especially the intermolecular distance *d*_1_.

### Spin-polarized calculations

For the spin-polarized calculations, the structural optimization is started with three different initial atomic spin configurations as indicated in Fig. 1(b), i.e., non-magnetic and anti-ferromagnetic 1 and 2 with *C2/m* symmetry. The structures are then relaxed without a symmetry restriction using a spin-polarized electronic calculation. We use the experimental data measured at 17.5 GPa^8^ as the initial structure for the optimization at 10 GPa. The pressure step size is 10 GPa. The optimized structures are divided into ferrimagnetic and anti-ferromagnetic structures. The ferrimagnetic structures are similar to the anti-ferromagnetic arrangement but have unequal magnetic moments. The ferrimagnetic configuration agrees well with the *C2/m* symmetry of the unit cell, which has three inequivalent atoms: four O1 atoms, two O2 atoms and two O3 atoms. Four O1 atoms are located on the diagonal of the (O_2_)_4_ structure. The spins of these four O1 atoms are parallel and equal, while the spins of the O2 and O3 atoms are anti-parallel to those of the O1 atom and not equal to those of the O1 atoms. The anti-ferromagnetic spin arrangement agrees with *Fmmm* symmetry in which all atoms are equivalent.

**Figure 6:**
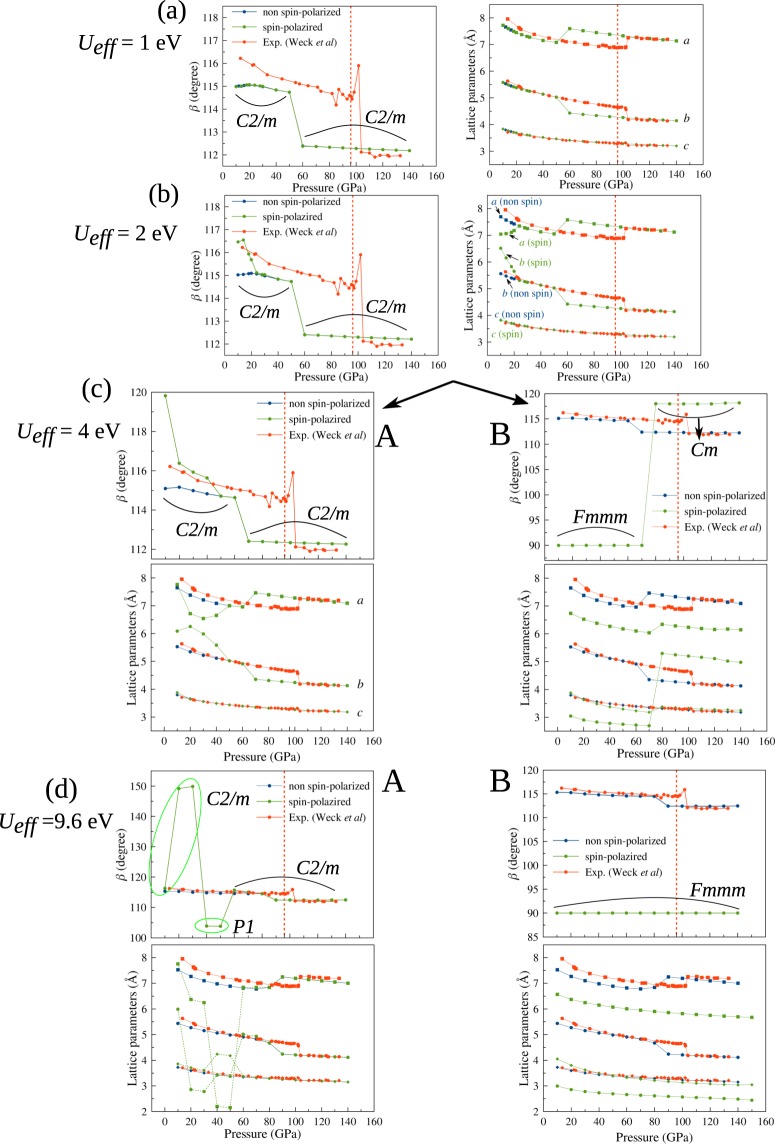
(**a**–**d**) Optimized lattice parameters *β, a, b, c* for groups A and B calculated with *U*_*eff*_ = 1, 2, 4, and 9.6 eV, respectively. The initial configurations before optimization were *C2/m* non-magnetic and *C2/m* anti-ferromagnetic 1, as shown in Fig. 1(b). The difference between the two groups appears at *U*_*eff*_ > 2 eV.

First, for simplicity, we discuss the GGA + vdW-D + U calculation with a constant *U*_*eff*_. In Fig. 6(a–d), we show the optimized lattice parameters *β, a, b*, and *c* for the initial spin configuration of groups A and B calculated with *U*_*eff*_ = 1, 2, 4, and 9.6 eV, respectively. The initial spin configurations are either *C2/m* non-magnetic or *C2/m* anti-ferromagnetic, as shown in Fig. 1(b). With *U*_*eff*_ = 1 eV, there is no difference between groups A and B in the spin-polarized calculation and in the non-spin-polarized calculations. All calculations predict non-magnetic properties for the epsilon phase. When *U*_*eff*_ increases to 2 eV, the low-pressure magnetic epsilon phase appears in the pressure range from 10 GPa to 20 GPa in both groups A and B. The absolute magnetization collapses when the pressure becomes higher than 20 GPa. Then, the epsilon phase changes to a high-pressure non-magnetic phase, and at 50 GPa, it transforms into the zeta phase. Moreover, the range of the low-pressure magnetic epsilon phase increases as *U*_*eff*_ increases: from 10–20 GPa with *U*_*eff*_ = 2 eV to 10–50 GPa with *U*_*eff*_ = 4 eV in group A or 10–70 GPa with *U*_*eff*_ = 4 eV in group B (Fig. 6(b,c)). In a neutron diffraction experiment^12^, the magnetization was found to collapse at ~7–8 GPa at 1.4 ~ 4 K.

When *U*_*eff*_ increases to 4 and 9.6 eV, the optimized structures retain the *C2/m* symmetry in group A or change to *Fmmm* symmetry in group B. With *U*_*eff*_ = 4 eV, the epsilon phase in group B is predicted as an *Fmmm* anti-ferromagnetic structure from 10 GPa to 70 GPa. After 70 GPa, it transforms to a non-magnetic *Cm* phase, which also has semiconductor characteristics. The symmetry in group A is *C2/m* for both the magnetic and non-magnetic phases. As *U*_*eff*_ increases to 9.6 eV, the epsilon phase is predicted to have either *C2/m* or *P1* symmetry in group A and *Fmmm* anti-ferromagnetic order in group B. In groups A and B, the enthalpies of the magnetic structures are always lower than those of the non-magnetic structures before the epsilon-zeta transition occurs. Details of the enthalpy and magnetization of groups A and B are described in the Supplementary Materials.

**Figure 7:**
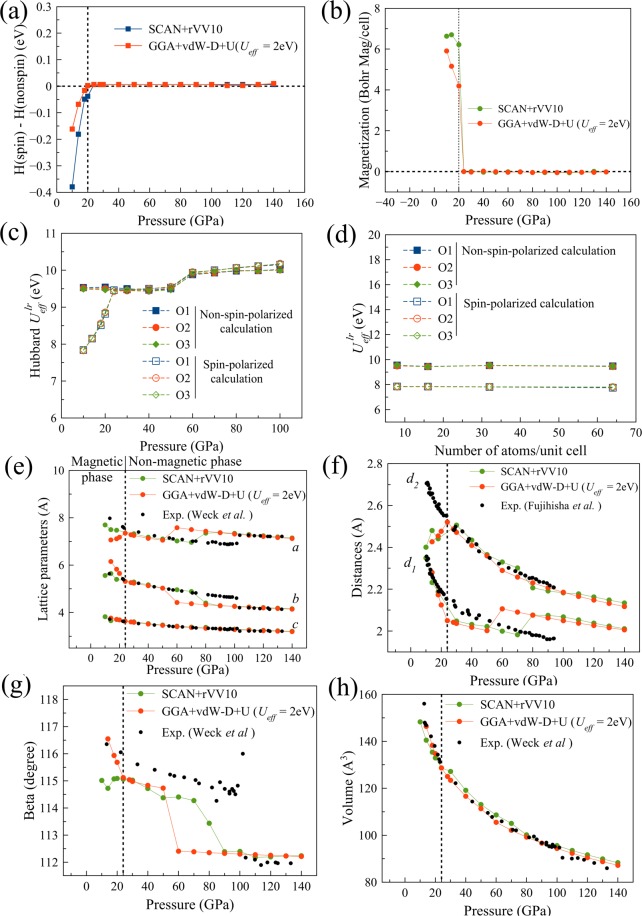
(**a**) The difference in enthalpy between the spin-polarized and non-spin-polarized calculations for the SCAN + rVV10 and GGA + vdW-D + U (with *U*_*eff*_ = 2 eV) methods; (**b**) the absolute magnetization collapses at 20 GPa for both the SCAN + rVV10 and GGA + vdw-D + U (*U*_*eff*_ = 2 eV) methods; the dependence of *U*^*lr*^_*eff*_ on compression (**c**) and the unit cell size (**d**) in the non-spin-polarized and spin-polarized calculations; (**e**–**h**) the optimized lattice parameters a, b, c and *β* and the intermolecular distances *d*_*1*_ and *d*_*2*_ and unit cell volume for the magnetic phase (below 20 GPa) and non-magnetic phase (above 20 GPa).

Finally, we perform a spin-polarized SCAN + rVV10 calculation for a comparison with the GGA + vdW-D + U calculation. Figure 7(a) shows the difference in enthalpies between the spin-polarized and non-spin-polarized calculations for the SCAN + rVV10 method and the GGA + vdW-D + U method at *U*_*eff*_ = 2 eV. With both methods, the magnetic phase is predicted to be more stable than the non-magnetic phase up to 20 GPa. The spin-polarized SCAN + rVV10 calculation also predicts ferrimagnetic order. The magnetization collapses at 20 GPa (Fig. 7(b)). The result from the SCAN + rVV10 calculation is consistent with the that of the GGA + vdW-D + U calculation at *U*_*eff*_ = 2 eV, where the ferrimagnetic phase is also predicted up to 20 GPa. This suggests that *U*_*eff*_ = 2 eV is a suitable value for epsilon-oxygen at low pressures (below 20 GPa).

In Fig. 7(c,d), we investigate the dependence of *U*^*lr*^_*eff*_ calculated from the first-principles linear response method for the non-magnetic and magnetic structures with respect to compression (Fig. 7(c)) and the unit cell size at 10 GPa (Fig. 7(d)). We use the structures from either the non-spin-polarized optimization or spin-polarized optimization with fixed *U*_*eff*_ = 2 eV to calculate the value of *U*^*lr*^_*eff*_ in compression. The difference in the value of *U*^*lr*^_*eff*_ obtained from the linear response method in non-spin-polarized and spin-polarized calculations shows how the conventional linear response method predict for the non-magnetic and magnetic structures. As we can see from Fig. 7(c) that the first-principles values of *U*^*lr*^_*eff*_ for the magnetic structures are still high (~8 eV) but, interestingly, smaller than those for the non-magnetic structures (~9.6–10.2 eV) at pressures below 20 GPa. The difference becomes almost zero at pressures above 20 GPa, where the enthalpy comparison shows that the structures are non-magnetic. We also check the size dependence of *U*^*lr*^_*eff*_ at 10 GPa, as shown in Fig. 7(d). The values of *U*^*lr*^_*eff*_ virtually do not depend on the size of the unit cell in both cases with and without spin polarization. The limitation of the linear response method in accounting for the screening effect of the opposite spin channel of the same site was demonstrated in a recent paper^30^. This may be a reason why the linear response method applied in this study predicts *U*^*lr*^_*eff*_ ~ 9.6 eV at pressures below 20 GPa. Another posibility is that we calculated the self-consistent *U* based on the GGA functionals which is less accurate than SCAN. Recent study of transition metal monoxides^31^ shows that the SCAN + vdW + U with the self-consistent *U* calculated based on SCAN predicts good ground state of FeO.

Figure 7(e–h) shows a comparison between the spin-polarized SCAN + rVV10 calculation, the GGA + vdW-D + U calculation and the experiments. The lattice parameters *a, b*, and *c* predicted from the SCAN + rVV10 calculation are more accurate than those from the GGA + vdW-D + U calculation, but the *β* value is less accurate, especially at pressures below 20 GPa. Overall, at low pressure (10 ~ 20 GPa), our calculations suggest the existence of a magnetic epsilon phase at 0 K. Our results also suggest that the value of the Hubbard U parameter at low pressure should be approximately 2 eV, which is much smaller than 9.6 eV predicted by the first-principles linear response method.

It should be mentioned that the optimized structures in spin-polarized SCAN + rVV10 can be either *P1* or *C2/m* symmetric for 8-atom primitive unit cell. But the lattice parameters of the two structures are very close including *d*_1_ and *d*_2_. Therefore in Fig. 7(e–h), we only show the data of *C2/m* structures. The structures for the non-magnetic phase are always *C2/m* symmetric.

As for the intermolecular distances *d*_1_ and *d*_2_, the spin-polarized SCAN + rVV10 method shows an improvement in the calculation of the *d*_1_ closer to the experimental value, but the *d*_2_ is underestimated. The behaviour is similar to that of the spin-polarized GGA + vdW-D + U method as shown in Fig. 7(f). In the next section, we use a larger unit cell in geometry optimization to solve the problem of the underestimations of the *d*_1_*, d*_2_.

### SCAN + rVV10 geometry optimization with a 16-atom supercell (conventional unit cell)

**Figure 8:**
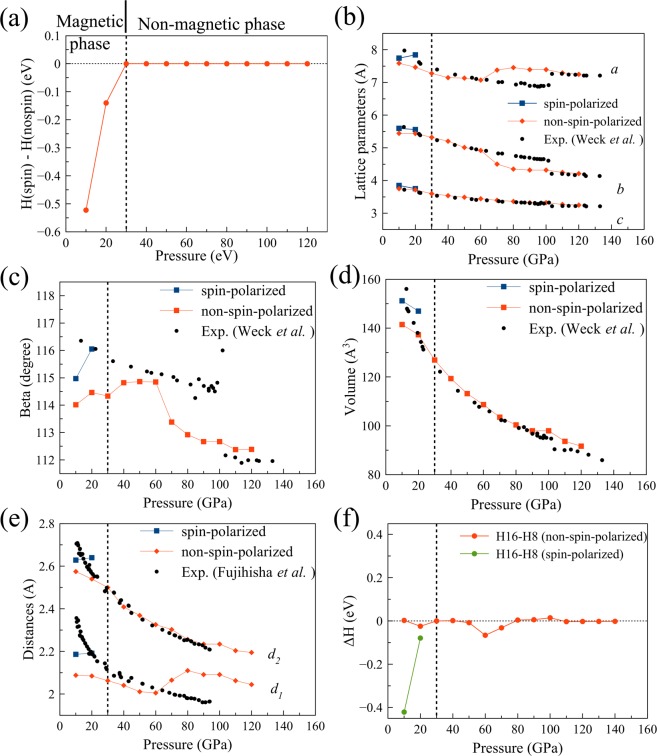
(**a**) The differences in the enthalpy, (**b**) lattice parameters, (**c**) beta, (**d**) volume of the unit cell, (**e**) the intermolecular distances *d*_*1*_ and *d*_*2*_ between the spin-polarized and non-spin-polarized SCAN + rVV10 calculations with a 16-atom conventional unit cell; (**f**) The difference in enthalpy between a 16-atom conventional unit cell and an 8-atom primitive cell.

In the previous sections, we consider a primitive unit cell consisting of 8 atoms. In this section, we investigate a larger unit cell that includes 16 atoms. The structures of the two unit cells are shown in Fig. 1(a,b). A 16-atom conventional unit cell provides more degrees of freedom for the oxygen atoms to relax. Therefore, we expect that the optimization can reach a more stable state. In this section, we only discuss the results from the SCAN + rVV10 calculation. As shown in Fig. 8(a), the magnetic phase is still predicted at low pressure (with 16-atom unit cell, the magnetization collapses at 30 GPa). Above 30 GPa, both the spin-polarized and non-spin-polarized SCAN + rVV10 calculations predict non-magnetic structures.

For the optimizations of primitive 8-atom unit cell, the *d*_1_ is closed to the measurement while the *d*_2_ is underestimated. The optimized structures of the 16-atom unit cell can be either *P*1 symmetry or *C*2*/m* symmetry. The enthalpy of the *P*1 structure is about 0.06 eV/atom lower than that of the *C*2*/m* structure. And the lattice parameters of *P1* structure are similar to those of primitive 8-atom unit cell, which means the *d*_*1*_ is closed to the measurement while the *d*_*2*_ is underestimated. Therefore we do not report the lattice parameters of the *P*1 structure. The lattice parameters of the *C*2*/m* structure of the 16-atom unit cell are shown in Fig. 8(b–e). The magnetic structures at 10 and 20 GPa have more accurate lattice parameters than the non-magnetic structures. Especially, the intermolecular distances *d*_1_ and *d*_2_ are improved much. In Fig. 8(f), we compare the enthalpy of the 16-atom structure and the 8-atom structure with magnetic configuration (spin-polarized calculation) and with non-magnetic configuration (non-spin-polarized calculation). The 16-atom unit cell with the magnetic order is the most stable and the lattice parameters are also the most consistent with the measurements.

## Summary and Conclusion

The crystal, electronic and magnetic structures of solid oxygen in the epsilon phase have been investigated using the SCAN + rVV10 method and the GGA + vdW-D + U method. The spin-polarized SCAN + rVV10 method with an 8-atom model provides lattice parameters consistent with the experimental results over the entire pressure range except for the intermolecular distances *d*_1_ and *d*_2_. When the size of the unit cell is extended to 16 atoms, the agreement between the intermolecular distances and the experimental values is greatly improved. Therefore, the SCAN + rVV10 method with a conventional 16-atom unit cell is the most suitable model for the epsilon phase of solid oxygen. The spin-polarized SCAN + rVV10 models predict a magnetic phase at low pressure. Since the lattice parameters of the predicted magnetic structure are consistent with the experimental lattice parameters measured at room temperature, our results may suggest that the epsilon phase is magnetic even at room temperature.

It is important to note that no ad hoc parameters are required in the SCAN + rVV10 calculation. The GGA + vdW-D + U (with an ad hoc value of *U*_*eff*_ = 2 eV at low pressure instead of the first-principles value *U*^*lr*^_*eff*_ ~ 9 eV)^13^ and hybrid functional methods^14^ provide similar results to the SCAN + rVV10 method; however, they do not provide reasonable values for the intermolecular distances. Recent study of transition metal monoxides^31^ shows that the SCAN + vdW + U with the self-consistent U calculated based on SCAN predicts good ground state of FeO. The SCAN + vdW + U may be a good choice for the calculation of solid oxygen’s ground state.The possibilities of further improving the result of the SCAN + rVV10 calculation are discussed in the Supplementary Materials.

## Supplementary information

First-principles calculations of the epsilon phase of solid oxygen
