# Supplementary material for: First-principles calculations of the epsilon phase of solid oxygen
Source: Sci Rep. 2019 Jun 19;9:8731. doi: 10.1038/s41598-019-45314-9 (PMC6584638; doi:10.1038/s41598-019-45314-9)
Supplement: Supplementary file 1 — First-principles calculations of the epsilon phase of solid oxygen [file 41598_2019_45314_MOESM1_ESM.pdf]

Supplementary Information

First-principles calculations of the epsilon phase of solid oxygen

Le The Anh[1,3*], Masahiro Wada[2], Hiroshi Fukui[2], Tsutomu Kawatsu[1], and Toshiaki Iitaka[1]

[1]Computational Astrophysics Laboratory, RIKEN, 2-1 Hirosawa, Wako, Saitama 351-0198, Japan

[2]Graduate school of Material Science, University of Hyogo, 3-2-1 Kouto, Kamigori, Hyogo, 678-1297, Japan

[3]Centre for Computational Physics, Institute of Physics, Vietnam Academy of Science and Technology, 10 Dao Tan, Ba Dinh, Hanoi, Vietnam

Email: letheanh102@gmail.com

Supplementary Information

First-principles calculations of the epsilon phase of solid oxygen

Le The Anh[1,3*], Masahiro Wada[2], Hiroshi Fukui[2], Tsutomu Kawatsu[1], and Toshiaki Iitaka[1]

1. Lattice parameters calculated with different DFT functionals

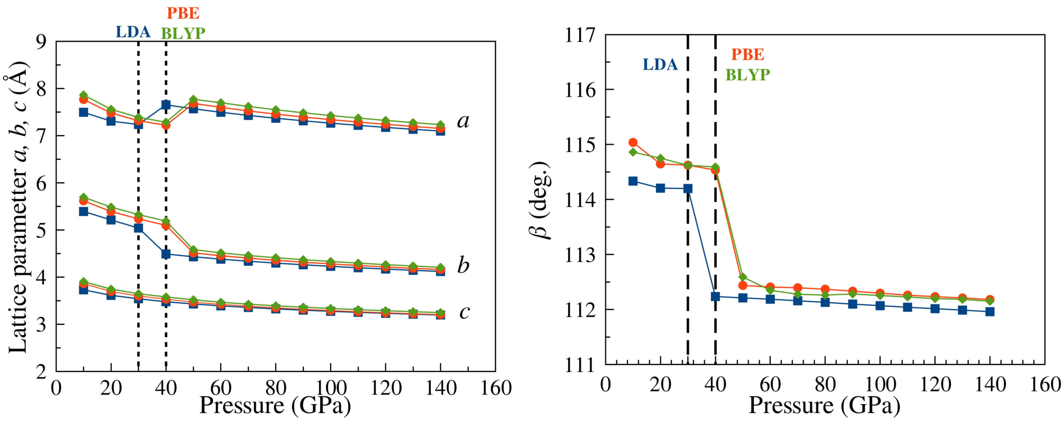

Supplementary Figure S1. Lattice parameters *a, b,* and *c* and the angle *β* calculated with the LDA, PBE and BLYP functionals.

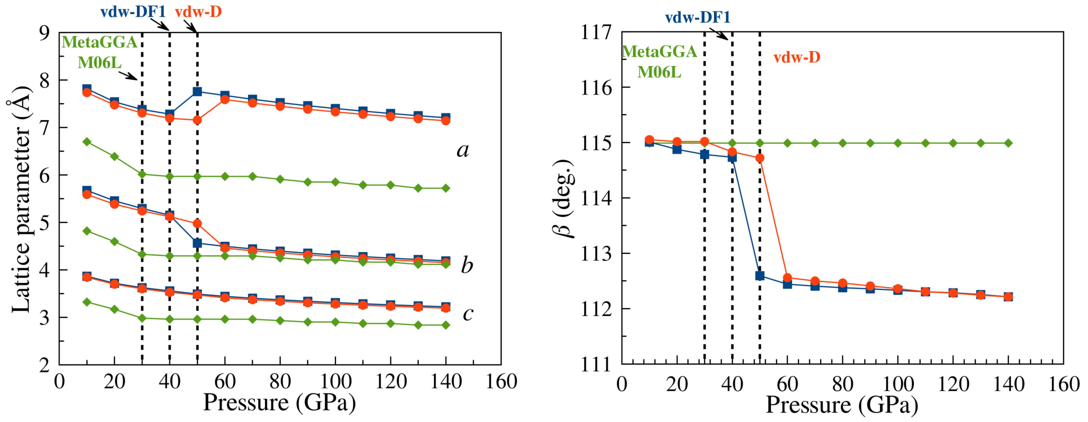

Supplementary Figure S2. Lattice parameters *a, b,* and *c* and the angle β calculated with the meta-GGA (M06L)[1], GGA+semi-empirical van der Waals[2] (vdW-D) and van der Waals functional (vdW-DF1)[3] methods.

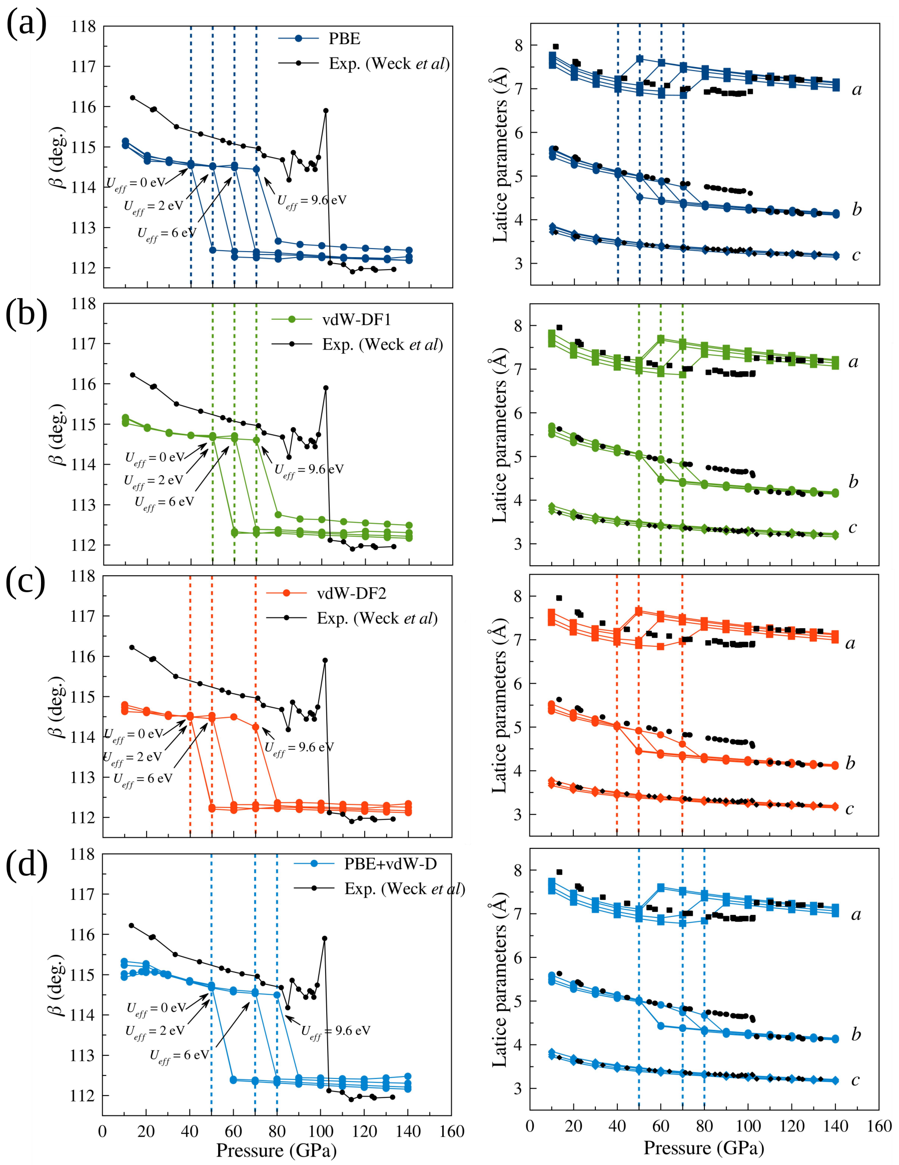

Supplementary Figure S3. Lattice parameters β*, a, b,* and *c* calculated with the GGA (PBE), vdW-DF1 functionals[3], revised vdW-DF2 functionals[7], and the semi-empirical GGA+vdW-D[2] method. The value of U_{eff} was changed from 0 eV to 9.6 eV.

In this Supplement, we compare the lattice parameters *a, b,* and *c* and the angle β calculated with different methods. Figure S1 shows the results from the LDA, PBE, and BLYP functionals. Figure S2 shows the data calculated with meta-GGA (M06L)[1], the van der Waals functional[3] (vdW-DF1) and the GGA + semi-empirical van der Waals[2] (vdW-D) method. Figure S3 shows the lattice parameters calculated with the GGA (PBE), van der Waals functionals vdW-DF1[3] and revised van der Waals functionals vdW-DF2[7], and the semi-empirical GGA+vdW-D[2] method. The value of U_{eff} was changed from 0 eV to 9.6 eV.

2. Enthalpy and magnetization of optimized structures calculated from the spin-polarized GGA+vdW-D+U method

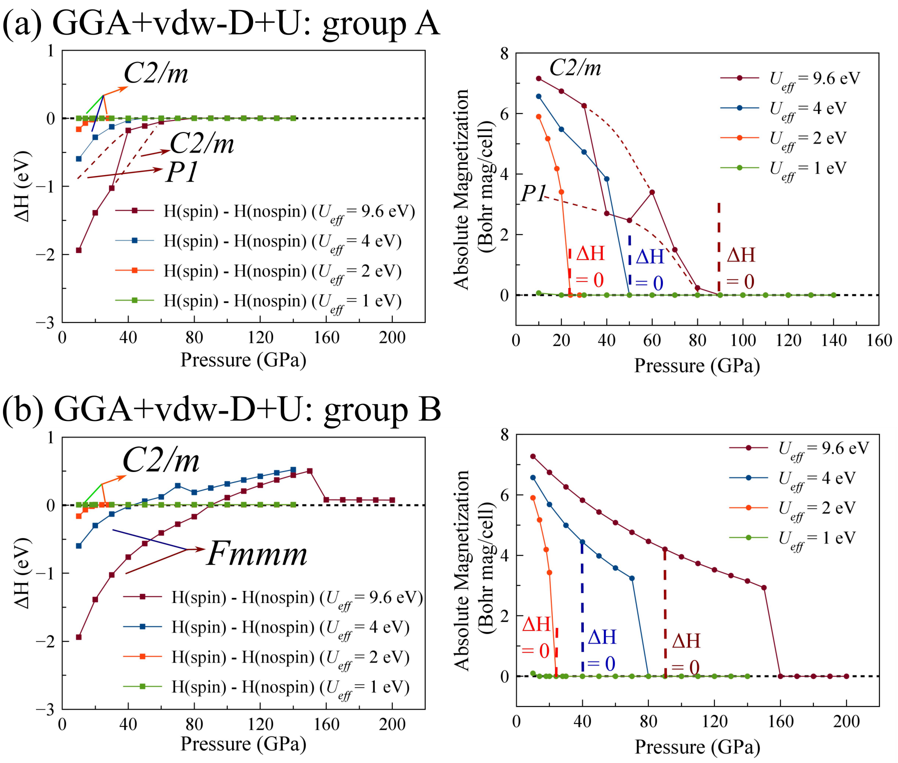

Supplementary Figure S4. The difference in enthalpies between the spin-polarized and non-spin-polarized calculations and absolute magnetizations calculated for (a) group A and (b) group B at U_{eff} = 1, 2, 4, and 9.6 eV.

In Figure S4(a-b), we compare the enthalpies of optimized structures obtained from the non-spin-polarized calculations and spin-polarized calculations for groups A and B, respectively. The difference in enthalpy can be considered as the magnetic energy. Because we compare groups A and B with non-spin-polarized structures, the zero energies are the same for groups A and B. The absolute magnetizations are shown on the right-hand side. In groups A and B, the enthalpies of the magnetic structures are always lower than those for the non-magnetic structures before the epsilon-zeta transition occurs. The structures of group A are as stable as those of group B at low pressure. Moreover, the range of the low-pressure magnetic epsilon phase increases as U_{eff} increases. This means that the Hubbard U not only enhances the localization of the electron but also stabilizes the magnetic structure.

3. Molecular dynamics simulation

Figure S5 shows the radial distribution function of the O-O distance g(r) simulated for the structure at 10 GPa and at 5 K and 300 K. The time step is 0.5 fs. We used the NpT ensemble with

a Langevin thermostat, which was implemented in the VASP code[9]. The functional function was PBE without vdw-D and Hubbard U. The structures were simulated at a constant pressure of 10 GPa and constant temperatures of 5 K and 300 K. The structure consisted of 16 oxygen atoms in a conventional unit cell. Our non-spin-polarized PBE molecular dynamics (MD) simulations at 5 K show that d_1 in the MD simulation is extended from 2 Å to 2.2 Å, which is closer to the measurement value at 2.35 Å. The MD simulation at 300 K shows a broadening distribution of d_1 varying from 2 Å to 2.2 Å. This preliminary result suggests that there may be many meta-stable structures co-existing at low pressure and that the d_1 values obtained from the measurements were an average value of these meta-stable structures. The MD simulation, thus, is more suitable to find a global minimum of solid oxygen than a conventional geometry optimization. Further spin-polarized molecular dynamics simulations will be carried out in the next study.

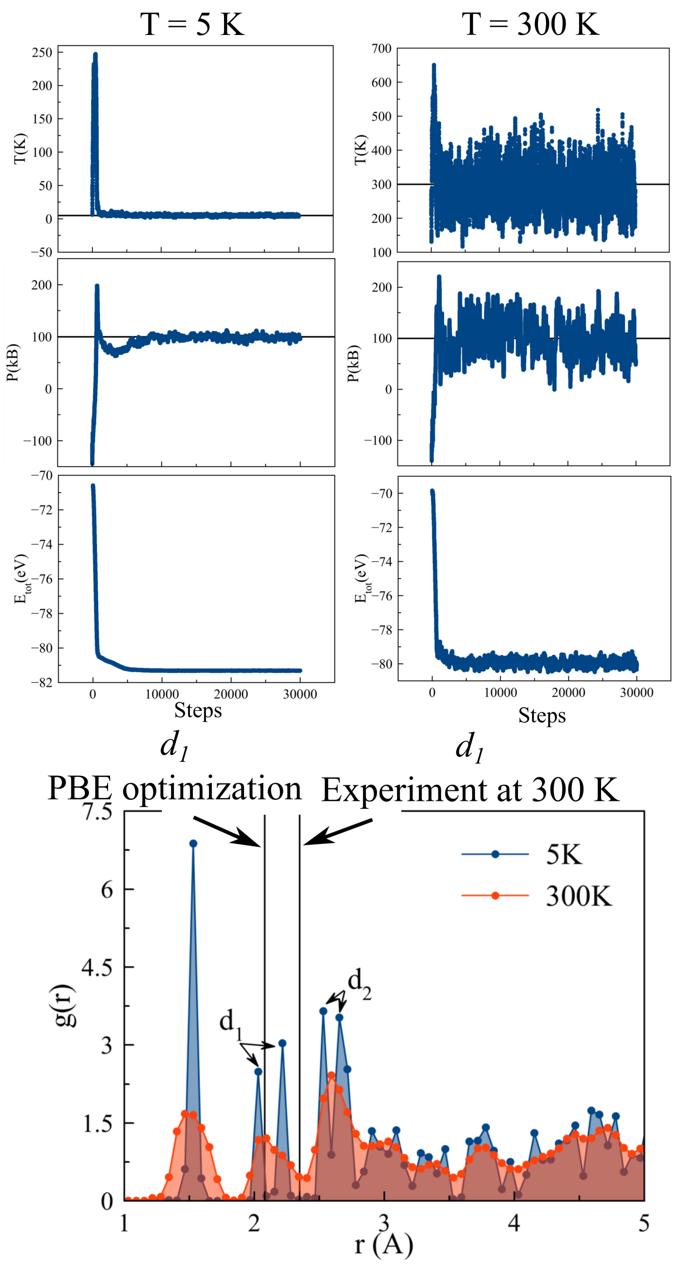

Supplementary Figure S5. Radial distribution function of the O-O distance g(r) simulated for the structure at P = 10 GPa with T = 5 K and 300 K. The time step is 0.5 fs.

4. More sophisticated methods

Finally, the poor description of DFT in many-body interactions[5,8] should be considered. In reference[8], the authors used a multiconfigurational complete active space second-order perturbation (CASPT2) to estimate the singlet state of an $(O_2)_4$ cluster and obtained reasonable results for d_1 and d_2. However, a similar calculation for a periodic structure is still missing. Other studies suggest the necessity of a multi-reference wave function[5] or a van der Waals density functional with spin-polarized-dependent gradient correction[4, 6]. Higher levels of calculation beyond the meta-GGA may be a key to solving this problem. Recent study of transition metal monoxides[10] shows that the SCAN+vdW+U with the self-consistent U calculated based on SCAN predicts good ground state of FeO. The SCAN+vdW+U may be a good choice for the calculation of solid oxygen's ground state.

References

1. Yan Zhaoa and Donald G. Truhla, A new local density functional for main-group thermochemistry, transition metal bonding, thermochemical kinetics, and noncovalent interactions, *J. Chem. Phys.* **125**, 194101 (2006).

2. S. Grimme, Semiempirical GGA-type density functional constructed with a long-range dispersion correction, *J. Comp. Chem.* **27 (15),** 1787-1799 (2006).

3. M. Dion, H. Rydberg, E. Schroder et al., Van der Waals Density Functional for General Geometries, *Phys. Rev. Lett.* **92**, 246401 (2005)

4. Edward B. Linscott, Daniel J. Cole, Michael C. Payne, and David D. O'Regan, Role of spin in the calculation of Hubbard U and Hund's J parameters from first principles, *Phys. Rev. B* **98**, 235157 (2018).

5. L. Craco, M. S. Laad, and S. Leoni, Microscopic description of insulator-metal transition in high-pressure oxygen, *Sci. Rep.* **7**, 2632 (2017).

6. M. Obata, M. Nakamura, I. Hamada, and T. Oda, Improving the description of nonmagnetic and magnetic molecular crystals via the van der Waals density functional, *J. Phys. Soc. Jpn.* **84**, 024715 (2015).

7. I. Hamada, van der Waals density functional made accurate, *Phys. Rev. B* **89**, 121103(R) (2014).

8. M. Bartolomei, J. Perez-Rios, E. Carmona-Novillo, M. I. Hernandez, J. Campos-Martinez, and R. Hernandez-Lamoneda, Can density functional theory methods be used to simulate the epsilon phase of solid oxygen?, *Chem. Phys. Lett.* **592**, 170-174 (2014).

9. G. Kresse and J. Furthmüller, Efficiency of *ab-initio* total energy calculations for metals and semiconductors using a plane-wave basis set, *Comput. Mat. Sci.* **6 (1),** 15-50 (1996).

10. H. Peng and J. P. Perdew, Synergy of van der Waals and self-interaction corrections in transition metal monoxides, *Phys. Rev. B* **96**, 100101 (R) (2017).
